# Aerobiological Dynamics and Climatic Sensitivity of Airborne Pollen in Southeastern Türkiye: A Two-Year Assessment from Siirt

**DOI:** 10.3390/biology14070841

**Published:** 2025-07-10

**Authors:** Salih Akpınar

**Affiliations:** Department of Biology, Faculty of Science and Letters, Kafkas University, Kars 36100, Türkiye; slh_akpinar@hotmail.com; Tel.: +90-0554-755-09-22

**Keywords:** airborne pollen, pollen diversity, meteorological factors, Siirt, pollen calendar

## Abstract

This study investigates the composition, abundance, and seasonal variability of airborne pollen in Siirt, a transitional region between the Irano-Turanian and Mediterranean phytogeographical zones in southeastern Türkiye. The main objective was to assess pollen diversity and its relationship with meteorological parameters over a two-year period (2022–2023). Airborne pollen was collected using a Hirst-type volumetric pollen and spore trap; a total of 18,666 pollen grains/m^3^ belonging to 37 taxa were identified. Of these, 70.67% originated from woody taxa and 29.33% from herbaceous taxa. Peak concentrations occurred in April, with the lowest levels in December. The dominant taxa, all exceeding 1% of the total, were Pinaceae (31.00%); Cupressaceae/Taxaceae (27.79%); Poaceae (18.42%); Moraceae (4.23%); Amaranthaceae (2.42%); Urticaceae (2.13%); *Quercus* (1.55%); Fabaceae (1.29%); and *Rumex* (1.02%). Spearman’s correlation analysis revealed significant relationships between daily pollen concentrations and meteorological variables such as temperature, humidity, precipitation, and wind speed. These findings highlight that both climatic conditions and the surrounding vegetation, shaped by regional land cover, play a crucial role in determining pollen dynamics. In conclusion, this study provides the first aerobiological baseline for Siirt and contributes valuable data for allergy-risk forecasting and long-term ecological monitoring in southeastern Türkiye.

## Simple Summary

Pollen is a fine powder released by plants that can cause allergic reactions in sensitive people. Understanding how pollen changes throughout the year helps scientists to predict allergy seasons and protect public health. In this study, we collected daily pollen samples from the air in Siirt, a city in southeastern Türkiye, over two years. We recorded how much pollen was present, what types of plants it came from, and how it changed with the weather. Most of the pollen came from trees, like Pinaceae and Cupressaceae/Taxaceae, and it was most common in spring, especially in April. Poaceae pollen also lasted longer and was more common during summer. We found that temperature, rain, and humidity strongly affected how much pollen was in the air. These results show that both plants in the area and changing weather conditions influence pollen levels. This research provides the first detailed look at pollen in Siirt and will help to create local pollen calendars. These calendars are useful for doctors, allergy sufferers, and decision-makers for an understanding of when allergy risks are highest and how to plan for them.

## 1. Introduction

Pollen grains are biological structures that carry the male gametophytes of seed plants and play a critical role in plant reproduction [1]. In addition to their reproductive function, pollen grains are also important indicators in ecological and environmental studies such as biodiversity monitoring, paleobotanical reconstructions, and biogeographical assessments [2,3]. The scientific discipline of aerobiology, which focuses on airborne pollen and spores, provides essential data for understanding ecological patterns and assessing public health risks [4,5]. Numerous studies have shown that increased concentrations of allergenic pollen can trigger respiratory disorders, such as allergic rhinitis, asthma, and conjunctivitis, especially in sensitive individuals [5,6]. These conditions not only impair quality of life but also impose significant economic burdens on healthcare systems [7].

The intensity, diversity, and duration of airborne pollen are largely shaped by the phenological behavior of plants and are strongly influenced by climatic parameters such as temperature, precipitation, relative humidity, and wind speed [8,9]. In particular, wind-pollinated (anemophilous) taxa produce large quantities of lightweight pollen that can be transported over long distances, which is why they dominate atmospheric pollen assemblages [10]. Short-term meteorological fluctuations can significantly alter pollen production and atmospheric concentrations. For instance, increased temperatures often accelerate flowering and pollen release, whereas rainfall and high humidity may suppress these processes by inhibiting pollen dispersal or promoting sedimentation [8,11,12].

Aerobiological research in Türkiye has gained momentum in recent decades. Studies have been conducted in various biogeographic regions, including the Black Sea (Sinop), Central Anatolia (Konya), Eastern Anatolia (Kars), Southeastern Anatolia (Mardin), the Aegean (İzmir-Buca), Mediterranean (Hatay), and Marmara (İstanbul) [13,14,15,16,17,18,19]. These studies have shown that the composition and seasonal dynamics of atmospheric pollen vary significantly depending on local flora, elevation, land use, and climatic conditions. For example, *Betula* and *Fagus* dominate in humid regions, while Poaceae and Cupressaceae are more prevalent in drier steppe and montane zones [20].

Siirt Province is located in southeastern Türkiye, at the intersection of the Irano-Turanian and Mediterranean phytogeographical regions; it harbors rich floristic diversity and topographical heterogeneity [21]. Despite this ecological significance, no comprehensive aerobiological data have been reported for Siirt to date. Therefore, the present study aims to analyze the composition, abundance, and seasonal variation of airborne pollen in the city of Siirt during the years 2022–2023 and to examine the relationship between pollen concentrations and meteorological parameters.

## 2. Materials and Methods

### 2.1. The Study Area, Flora, and Climate Characteristics

**Figure 1:**
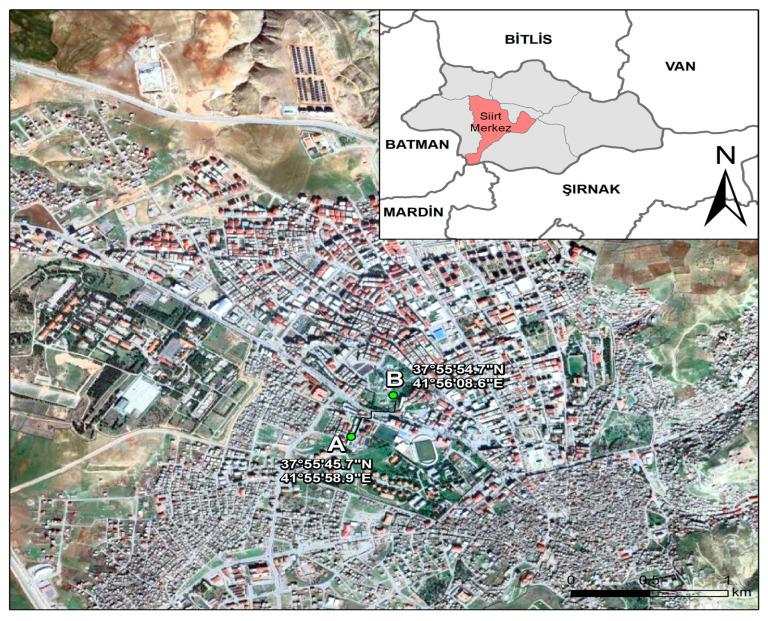
Location map of Siirt: (**A**) sampling station; and (**B**) weather station.

Siirt Province is located in southeastern Türkiye, at the intersection of the Irano-Turanian and partially Mediterranean phytogeographic regions, positioned at 37°55′38″ N latitude and 41°56′31″ E longitude. The total surface area of the province is 6186 km^2^; its average elevation is approximately 895 m above sea level. The study area is bordered by Bitlis to the north, Batman to the west, Mardin and Şırnak to the south, and Van to the east (Figure 1) [22].

This transitional position and diverse topography are among the main factors contributing to the province’s floristic richness [21]. Based on a literature review and field surveys conducted between 2018 and 2019, a total of 875 plant taxa belonging to 88 families and 397 genera were identified within the borders of Siirt Province [21]. Topographic variability, including high plateaus, valleys, and mountainous areas, directly influences microclimatic conditions and vegetation zones in the region [23]. The natural vegetation is largely composed of steppe formations and degraded forest remnants. Dominant woody species in these areas include oak (*Quercus*); willow (*Salix*), poplar (*Populus*);Turkish pine (*Pinus brutia*); birch (*Betula*); walnut (*Juglans regia*); plane (*Platanus*); maple (*Acer*); ash (*Fraxinus*); and various sclerophyllous shrubs [21,23].

**Figure 2:**
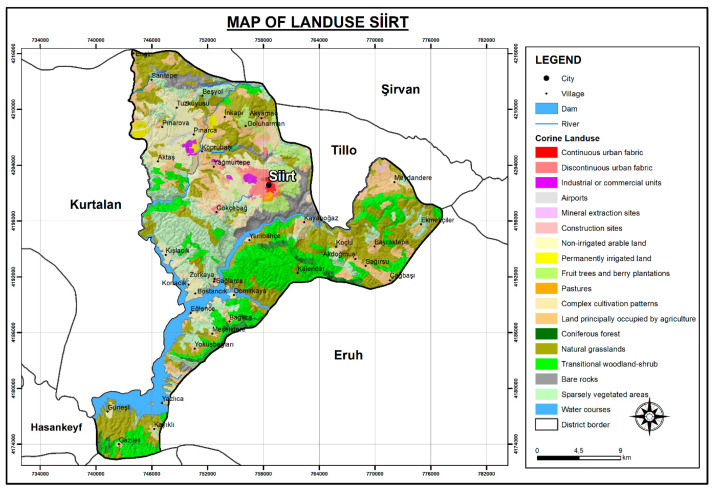
Land use map of Siirt. X-axis and Y-axis represent the geographical coordinates (latitude and longitude, in degrees).

To provide an ecological context for airborne pollen composition in Siirt, land cover data were derived from the CORINE (Coordination of Information on the Environment) Land Cover 2018 data. The analysis revealed that natural grasslands constitute the most extensive land cover class, accounting for 25.71% of the total area. These grasslands serve as major sources of herbaceous pollen, particularly from Poaceae and other anemophilous taxa. Transitional woodland–shrub areas (16.40%) and sparsely vegetated areas (15.22%) reflect degraded forest structures and open habitats, both contributing to pollen diversity through a mix of woody and herbaceous elements. Non-irrigated arable lands (12.09%) and agricultural areas with natural vegetation (11.95%) support both cultivated species and ruderal taxa, enhancing heterogeneity in pollen input. These dominant land cover types surrounding the sampling site offer important insight into potential pollen sources and help to explain the seasonal and taxonomic variability observed in the airborne pollen assemblage (Figure 2).

Siirt has a continental climate characterized by hot and dry summers and cold and wet winters. Rainfall is significantly reduced between June and October. According to long-term meteorological data spanning 52 years, the annual average temperature is 16.1 °C; the average maximum temperature is 21.8 °C; and the average minimum temperature is 11.1 °C. The annual average total precipitation is 692.0 mm. The highest recorded temperature is 46.0 °C, while the lowest is −15.6 °C.

**Figure 3:**
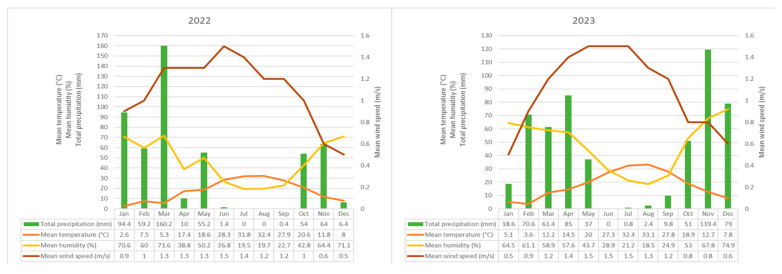
Meteorological parameters recorded at the meteorological station closest to the sampling site.

The meteorological parameters recorded during the study years—including relative humidity, temperature, precipitation, and wind speed, are summarized in detail in Figure 3.

### 2.2. Aerobiological Sampling and Statistical Analysis

During the 2022–2023 observation period, airborne pollen monitoring in Siirt was conducted using a Hirst-type volumetric pollen and spore trap (Lanzoni VPPS 2010, Bologna, Italy). The device was installed on the rooftop of a centrally located building at an approximate height of 15 m above ground level (41°30′30″ N, 42°43′42″ E) (see Figure 1A for the trap location and Figure 1B for the meteorological station). Weekly adhesive-coated Melinex tapes were cut into seven equal segments in the laboratory to obtain daily slides. Pollen grains were counted at 2 mm intervals under a Leica light microscope (Wetzlar, Germany) at 400× magnification, with each interval corresponding to one hour. The results were calculated as pollen grains per cubic meter of air (pollen/m^3^), in accordance with protocols established by the Spanish Aerobiology Network (REA) (Córdoba, Spain) and international recommendations [24].

The main pollen seasons (MPS) were determined using the widely accepted 2.5–97.5% method introduced by Andersen [25]. According to this method, the start of the pollen season was defined as the day when the cumulative pollen sum reached 2.5% of the annual pollen integral (APIn); the end of the season was marked by the 97.5% threshold. This approach ensures a standardized framework for comparing pollen seasons across taxa and years, reducing the bias caused by isolated pollen events outside the main flowering period.

Threshold levels for airborne pollen concentrations that can provoke allergic reactions in sensitive individuals were based on reference values published by REA and related studies [24,26,27]. These levels were classified as low, moderate, or high, reflecting their potential clinical impact. Accordingly, the number of days falling within the moderate- and high-concentration categories was used to define allergy-risk periods during the study years.

In addition to raw pollen counts, percentage-based data were also utilized to characterize dominant taxa and compare these with other regional studies. This strategy helps to minimize variations due to local environmental conditions and sampling artifacts; it is especially useful in retrospective and multi-year phenological analyses [24,25,28]. In this context, a pollen calendar was also created to illustrate the seasonal distribution of dominant taxa [29]. Although Galán et al. (2007) [24] recommend using at least five years of data for such calendars to reflect long-term variability, the calendar in this study was constructed based on the available two-year dataset (2022–2023) to provide a phenological characterization of airborne pollen patterns in Siirt [24,29].

Meteorological parameters including daily mean temperature, relative humidity, wind speed, and total precipitation were obtained from the nearest weather station operated by the Turkish State Meteorological Service, located approximately 1.3 km from the sampling site (41°29′49″ N, 42°44′04″ E) (Figure 1). The relationship between daily pollen concentrations and meteorological variables was analyzed using Spearman’s correlation test, performed in SPSS version 20, with statistical significance set at *p* < 0.05.

## 3. Results

### 3.1. Pollen Concentrations and Groups

**Figure 4:**
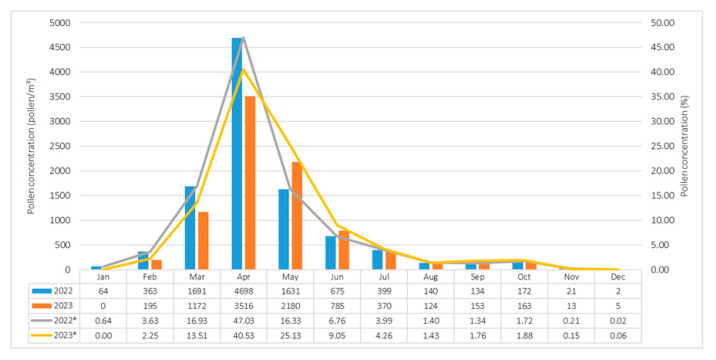
Monthly variation in airborne pollen concentrations in Siirt during 2022 and 2023. Values (%) represent annual mean percentages. Asterisks (*) indicate data from 2022 and 2023, respectively.

During the 2022–2023 observation period in Siirt, a total of 18,666 airborne pollen grains per cubic meter of air (pollen/m^3^) belonging to 37 taxa were identified. Among these, 20 taxa originated from woody plants, while 17 taxa were classified as herbaceous. Overall, 70.67% of the total airborne pollen was attributed to woody taxa, whereas 29.33% was contributed by herbaceous taxa. In 2022, a total of 9990 pollen grains/m^3^, representing 37 taxa were recorded; of these, 74.61% were attributed to woody taxa and 25.39% to herbaceous taxa. In 2023, 8676 pollen grains/m^3^ from 35 taxa were detected, with 70.67% attributed to woody taxa and 29.33% to herbaceous taxa (Table 1). The lowest monthly pollen concentrations in both years were recorded in December (2 pollen/m^3^), whereas peak levels occurred in April, reaching 4698 pollen/m^3^ in 2022 and 3516 pollen/m^3^ in 2023 (Figure 4).

### 3.2. Variations in Pollen Concentrations

During the 2022–2023 observation period, monthly fluctuations in airborne pollen concentrations were assessed in conjunction with APIn; these variations were found to be associated with meteorological parameters. Although airborne pollen was detected throughout the entire year in both 2022 and 2023, distinct differences in seasonal distribution patterns were evident.

**Figure 5:**
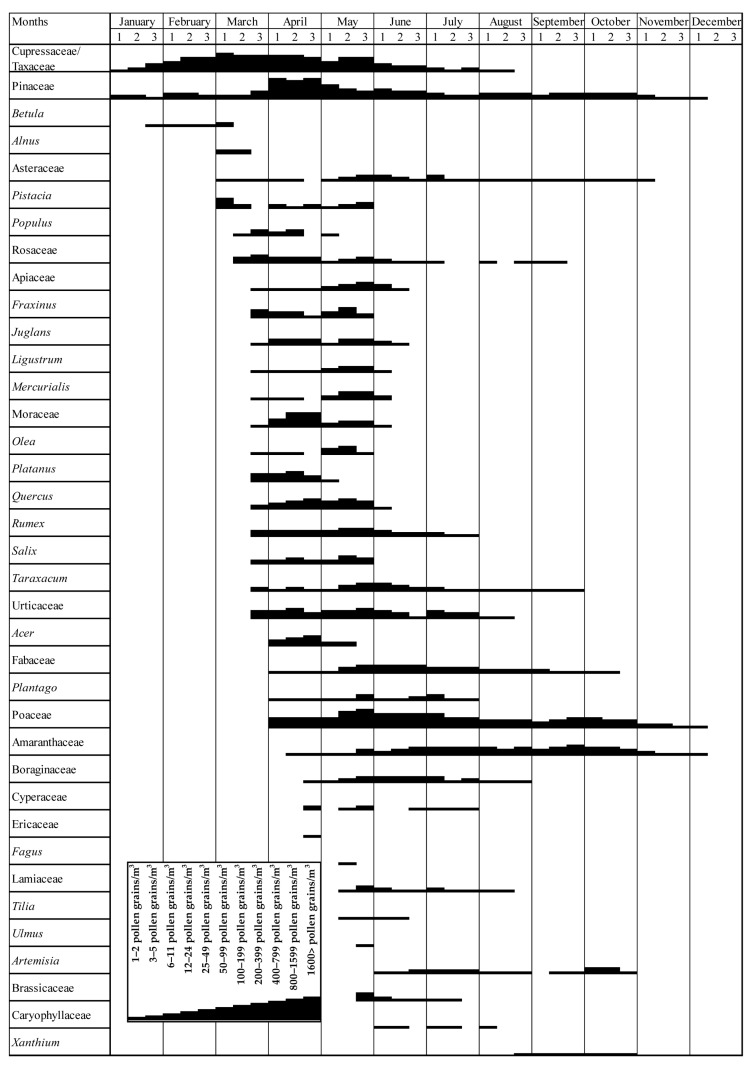
Pollen calendar for dominant taxa based on REA thresholds. Numbers 1, 2, and 3 indicate the first (days 1–10), second (days 11–20), and third (days 21–end) ten-day periods of each month, respectively.

In 2022, the peak pollen concentration was recorded in April, followed by March and May. This period coincided with a marked rise in temperature (March: 5.3 °C; April: 17.4 °C), while precipitation levels were high in March (160.2 mm) but decreased significantly in April (10 mm). These conditions suggest that elevated temperatures and reduced rainfall can enhance both pollen release and atmospheric dispersion. A pronounced decline in pollen concentrations occurred during the summer months, when temperatures ranged between 28–32 °C and precipitation was nearly absent. The lowest pollen levels were observed in December, characterized by low temperatures and high relative humidity (Figure 3 and Figure 5).

Similarly, in 2023, the highest pollen levels occurred in April, followed by May and March. In March, moderate temperatures (12.2 °C), decreased precipitation (61.4 mm), and relatively low humidity (58.9%) provided favorable conditions for pollen emission. Despite higher precipitation in April (85 mm), the temperature (14.5 °C) and humidity (57.6%) remained within optimal ranges for flowering, sustaining elevated pollen concentrations. In both years, a decline in pollen levels was observed during summer, driven by extreme heat and low rainfall. The minimum concentrations were again recorded in December, when temperatures dropped to 7–8 °C and relative humidity exceeded 70% (Figure 3 and Figure 5).

### 3.3. Pollen Calendar

Based on the two-year average dataset, the pollen calendar illustrates the MPS, intensity levels, and phenological distribution of dominant airborne taxa in the atmosphere of Siirt (Figure 5). According to the calendar, the earliest pollen taxa appeared during winter, primarily represented by woody taxa such as Cupressaceae/Taxaceae, Pinaceae, and *Betula*, which were detected from the second half of January. In February and March, additional woody taxa—including *Alnus*, *Quercus*, *Platanus*, and Moraceae—were recorded in the atmosphere. Notably, herbaceous taxa such as Urticaceae and Poaceae were also detected during this early season.

The period between late March and April marked the peak of pollen activity in terms of both diversity and concentration. During this interval, high levels of airborne pollen were recorded from a wide range of woody and herbaceous taxa, including Pinaceae, Poaceae, *Quercus*, *Juglans*, *Fraxinus*, *Plantago*, *Rumex*, Rosaceae, Fabaceae, Apiaceae, Amaranthaceae, and *Mercurialis*. Among these, Poaceae and Amaranthaceae exhibited prolonged presence in the atmosphere, extending into mid-summer due to their extended flowering periods. Notably, Pinaceae, Moraceae, and Cupressaceae/Taxaceae showed peak occurrences in April, when mean temperatures reached 17.4 °C in 2022 and 14.5 °C in 2023. This timing suggests that moderate spring temperatures play a critical role in triggering the flowering and pollen release of these taxa, thereby contributing to the pronounced peak observed during this period. (Figure 5).

Although total pollen concentrations decreased during summer, notable levels were still observed from late-summer and autumn-blooming herbaceous taxa such as *Artemisia*, *Xanthium*, Asteraceae, and Boraginaceae. In autumn, pollen from continuing herbaceous taxa, like Amaranthaceae, Poaceae, and Urticaceae, was still present, alongside late-flowering coniferous species such as *Cedrus*. The lowest pollen diversity and concentrations were recorded in November and December, when only a few pollen taxa were detected at minimal levels, indicating the end of the MPS and the onset of atmospheric dormancy (Figure 5).

### 3.4. Dominant Pollen Taxa and MPS Periods

Based on the two-year average data, nine airborne pollen taxa were identified as dominant in the atmosphere of Siirt, each contributing more than 1% to APIn. Among these, Pinaceae (31.00%), Cupressaceae/Taxaceae (27.79%), and Poaceae (18.42%) were the most abundant groups. Other dominant taxa included Moraceae (4.23%), Amaranthaceae (2.42%), Urticaceae (2.13%), *Quercus* (1.55%), Fabaceae (1.29%), and *Rumex* (1.02%). Collectively, these nine taxa accounted for approximately 89.94% of the total airborne pollen load across both study years (Table 1)

The longest MPS durations were recorded for Poaceae, spanning 187 days in 2022 and 174 days in 2023, followed by Urticaceae and Cupressaceae/Taxaceae. Conversely, the shortest MPS durations were observed for Moraceae (21 days in 2023) and *Quercus* (31 days in 2023) (Table 2).

The highest daily pollen concentrations were registered for Pinaceae, with peak values of 484 pollen/m^3^ on 3 April 2022, and 395 pollen/m^3^ on 7 April 2023. These findings indicate that woody taxa typically display short but intense pollen seasons, primarily in spring, while herbaceous taxa—particularly Poaceae and Amaranthaceae—tend to exhibit extended flowering periods and prolonged atmospheric presence (Table 2).

### 3.5. Meteorological Drivers of Daily Pollen Variability in Siirt (2022–2023)

The correlations between daily airborne pollen concentrations of dominant taxa and meteorological variables in Siirt were analyzed separately for the full years of 2022 and 2023, as well as for their respective MPS periods. The findings indicated that the influence of meteorological conditions on pollen levels was both taxon-specific and seasonally variable (Table 3).

Temperature exhibited differential effects across years. In 2022, Cupressaceae/Taxaceae and Moraceae showed negative correlations with temperature, whereas in 2023, stronger negative associations were detected for Pinaceae, Cupressaceae/Taxaceae, and Poaceae. Conversely, *Rumex* displayed a significant positive correlation with temperature in 2023, suggesting species-specific thermal responses (Table 3).

Relative humidity generally showed positive correlations with Cupressaceae/Taxaceae, Pinaceae, and Urticaceae, particularly during 2023. In contrast, Moraceae and Amaranthaceae exhibited negative associations with humidity. Among all variables, wind speed demonstrated the most consistent positive correlation, especially with Poaceae, across both years. Pinaceae and Fabaceae also responded positively to wind, whereas Moraceae exhibited a negative response (Table 3).

Precipitation generally showed weak or non-significant correlations with pollen concentrations; however, in 2023, significant negative associations were observed for Cupressaceae/Taxaceae and Pinaceae, indicating possible washout effects or rainfall-inhibited pollen release for these taxa (Table 3).

During the MPS periods, the role of meteorological drivers became more pronounced. Cupressaceae/Taxaceae, Amaranthaceae, and Urticaceae displayed positive correlations with temperature, whereas Pinaceae and Poaceae were negatively correlated. Relative humidity remained positively associated with Pinaceae, Cupressaceae/Taxaceae, and Urticaceae, while Moraceae and Amaranthaceae maintained their negative relationship. Wind speed continued to be positively correlated with Poaceae, followed by Pinaceae and Fabaceae. Although no strong associations were observed between precipitation and pollen concentrations during the MPS, several taxa demonstrated weak negative trends.

## 4. Discussion

This aeropalynological study, conducted in Siirt over two consecutive years (2022–2023), identified a total of 18,666 pollen grains/m^3^ belonging to 37 airborne taxa. Arboreal taxa exhibited a marked dominance, accounting for 70.67% of the total pollen load, compared to 29.33% for herbaceous taxa (Table 1). The airborne pollen spectrum in Siirt is shaped by its transitional location between the Irano-Turanian and partially Mediterranean phytogeographical zones, combined with its diverse topography and semi-arid climate. These environmental features, along with regional vegetation composition, significantly influence both the seasonal dynamics and taxonomic structure of atmospheric pollen.

Similar arboreal dominance has been reported in various regions of Türkiye, including Mardin (62.66%), Posof (72.3%), and to a lesser extent in Kars and Kars-Sarıkamış, where herbaceous taxa constitute a higher proportion, reaching up to 45–50% of the total pollen load [15,16,30,31]. At the international level, arboreal pollen is also dominant in cities such as Nicosia (68.2%) and Trieste (71%). Conversely, in oceanic and high-altitude environments—such as Funchal (Madeira) and Mexico City—herbaceous taxa often comprise over 50% of the annual pollen spectrum, reflecting differences in local flora and climate regimes [32,33,34,35].

The family Pinaceae—comprising genera such as *Pinus*, *Abies*, *Cedrus*, and *Picea*—is one of the most ecologically dominant conifer groups in temperate and boreal zones. These evergreen trees are well adapted to various environments, including montane areas and nutrient-poor soils, and play key roles in forest structure, carbon sequestration, and ecosystem resilience [36,37]. Evolutionary and genomic studies highlight their high phylogenetic coherence, adaptive plasticity, and conserved genome architecture, underscoring the central importance of Pinaceae in conifer biology [38,39].

Although Pinaceae produces substantial quantities of airborne pollen, it is generally considered to have low allergenic potential. Reported sensitization rates are relatively low, typically ranging from 1.5% to 6%, though regional variation exists [40]. Some studies have proposed that *Pinus radiata* and other species may function as adjuvants—exacerbating allergic responses triggered by more potent aeroallergens such as grasses [41,42]. Thus, while Pinaceae pollen is not a major allergen per se, it may indirectly contribute to the overall allergic burden by modulating immune responses.

In the present study, Pinaceae emerged as the most dominant airborne taxon in Siirt, comprising 31.00% of APIn (Table 1). Its MPS extended from early April to early August, with peak daily concentrations reaching 484 pollen grains/m^3^. These values are comparable to those observed in other southeastern Anatolian cities, such as Van (20.94%) and Elazığ (20–22%), where coniferous forests are prominent and *Pinus* species are either cultivated or naturally abundant [43,44]. Similarly, in Shiraz (Iran), Pinaceae was among the dominant spring taxa, contributing 15.11% to the total pollen load [45].

Internationally, high Pinaceae contributions have been reported in Trieste (Italy), where arboreal taxa account for over 70% of total atmospheric pollen, and in Nicosia (Cyprus), where Pinaceae and Cupressaceae together constitute more than 48% of the annual pollen spectrum [32,33]. In contrast, in humid Atlantic climates, such as Funchal (Madeira), Pinaceae and Cupressaceae together contribute only around 20% due to the predominance of mesophilic broadleaf vegetation [35]. These patterns underscore the influence of regional climate, elevation, and vegetation structure on Pinaceae pollen abundance and seasonality.

The families Cupressaceae and Taxaceae comprise ecologically important coniferous lineages distributed across temperate and Mediterranean biomes. Species of Cupressaceae, such as *Cupressus* and *Juniperus*, are ecologically adaptable and commonly occur in semi-arid ecosystems as well as in urban ornamental plantings. In contrast, Taxaceae—which includes genera such as *Taxus*—is typically associated with shaded understories of moist forests and characterized by slow growth and high shade tolerance [46,47]. Although phylogenetic studies confirm close evolutionary affinities between the two families; their distinct reproductive structures and ecological strategies justify their taxonomic separation [47,48,49].

Pollen from Cupressaceae and Taxaceae is recognized as a major contributor to winter and early-spring pollinosis. In particular, species of *Cupressus* are known for their prolific pollen production and strong allergenic potential. Allergenic components, such as Cup a 3, have been shown to elicit IgE-mediated responses in over 90% of sensitized individuals [50]. The extensive use of Cupressaceae in urban landscaping has been implicated in increasing human exposure and the emergence of “winter pollinosis,” especially in Mediterranean and semi-arid regions [51,52]. Sensitization rates can reach up to 9.3% in some populations; cross-reactivity with certain plant-derived foods may further complicate diagnosis and clinical management [53].

In the present study, Cupressaceae/Taxaceae ranked as the second most abundant pollen group in Siirt, contributing 27.79% to APIn (Table 1). The MPS extended from late February to late May, coinciding with rising spring temperatures. Similar early-season dominance of Cupressaceae has been documented in other southeastern Anatolian cities. For instance, it accounted for 50.86% of the total pollen in Hatay, peaking in February, and 27.79% in Mardin [16,18]. In contrast, its relative abundance was lower in Van (10.53%), a high-altitude region where colder conditions may limit conifer flowering [43]. In Shiraz (Iran), Cupressaceae was also an early-spring dominant taxon [45]. International reports from Nicosia (Cyprus) and Trieste (Italy) show high atmospheric concentrations of Cupressaceae, largely attributed to ornamental planting practices [32,33]. These patterns underscore the combined influence of native vegetation, urban landscaping, and regional climate in shaping the abundance and allergenic relevance of Cupressaceae/Taxaceae pollen.

The family Poaceae (grasses) is one of the most diverse and ecologically dominant angiosperm lineages, comprising more than 11,800 species distributed across approximately 780 genera worldwide [54,55]. Grasses serve as primary structural elements of steppe, savanna, and cultivated ecosystems, owing to their exceptional tolerance to drought, mechanical disturbance, and soil salinity [56,57]. Morphophysiological adaptations—such as C_4_ photosynthesis and the presence of salt-secreting glands—enhance their ecological plasticity and enable widespread success across both natural and anthropogenic habitats.

From an aerobiological and clinical perspective, Poaceae is among the most significant allergenic plant families. Grass pollen contains major IgE-binding proteins, such as Phl p 1 and Phl p 5, which are responsible for a substantial portion of seasonal allergic rhinitis and bronchial asthma cases worldwide [58,59]. Up to 40% of individuals with seasonal allergic symptoms are sensitized to grass pollen; immunological cross-reactivity with structurally similar food allergens can exacerbate clinical manifestations [60]. Environmental parameters, particularly high humidity and precipitation, enhance pollen hydration and fragmentation, thereby increasing airborne allergen release and symptom severity during peak seasons [61,62].

In Siirt, Poaceae emerged as the most dominant herbaceous taxon, accounting for 18.42% of the APIn load and exhibiting an extended pollen season from mid-April to mid-October (Table 1 and Figure 5). This prolonged flowering period and substantial abundance are consistent with findings from other semi-arid and steppe-affected regions in eastern Türkiye, including Bitlis (25.19%), Mardin (21.21%), and Elazığ (9.1%) [16,44,63]. In Shiraz (Iran), Poaceae was also identified as a major component of the pollen spectrum, strongly associated with spring temperatures, and contributing significantly to early summer peaks [45].

Comparable patterns have been documented internationally. In Salamanca (Spain), Poaceae constituted 21.4% of the total pollen spectrum. In Mexico City, values reached up to 30%, primarily due to the prevalence of both natural grasslands and urban green spaces [34,64]. The high concentrations and long seasonal persistence of grass pollen in Siirt suggest that Poaceae is likely one of the leading causes of pollinosis in the region, particularly from late spring through to early autumn.

The family Moraceae (mulberry family) encompasses approximately 1100 species across 37 genera, including *Ficus*, *Morus*, and *Artocarpus* [65,66]. These taxa are predominantly distributed in tropical and subtropical regions, but are also cultivated in temperate zones for their edible fruits, ornamental value, and ethnobotanical uses. In particular, species of *Morus* are widely planted in urban environments and along roadsides due to their rapid growth and dense canopies that provide ample shade [67,68].

*Morus* pollen is a clinically relevant aeroallergen associated with seasonal allergic rhinitis, bronchial asthma, and, in some individuals, oral allergy syndrome due to cross-reactivity with fruit proteins [67,69]. The widespread urban use of *Morus* species can lead to short-duration but high-intensity pollen exposure. Furthermore, environmental factors, such as increased humidity, can enhance the allergenic potential by promoting pollen grain rupture and allergen release [61,67].

In the present study, Moraceae ranked as the fourth most abundant airborne pollen taxon in Siirt, contributing 4.23% to APIn, with a distinct seasonal peak in April and May (Table 1 and Figure 5). Similar seasonal trends have been reported in Hatay, where Moraceae accounted for 7.2% of the annual pollen load and was identified among the dominant allergenic taxa, and in Mardin, where *Morus* pollen reached 6.19% [16,18]. Comparable observations have been made in La Plata (Argentina) and Köyceğiz (southwestern Türkiye), where short but intense *Morus* pollen seasons were associated with increased allergy consultations [70,71]. Although the flowering period of Moraceae is relatively brief, its high allergenic potential and rapid pollen accumulation make it a notable contributor to early spring allergy risk in Siirt.

The family Amaranthaceae comprises numerous species well adapted to arid, saline, and disturbed environments, especially in semi-arid regions. Genera, such as *Amaranthus* and *Salsola*, are prevalent in ruderal and steppe habitats and are characterized by ecological plasticity and extended flowering periods [72,73]. Pollen from Amaranthaceae is considered an important aeroallergen in dry climates and has been associated with allergic rhinitis and asthma, particularly during late summer and autumn, when exposure durations are prolonged [72,73].

In Siirt, Amaranthaceae accounted for 2.42% of the total airborne pollen load, exhibiting a prolonged season from late May through to late October (Table 1 and Figure 5). Similar late-season pollen patterns have been documented in Mardin, Hatay, and Elazığ, where members of this family are prominent contributors to the allergenic load [16,18,44]. In Shiraz (Iran), Amaranthaceae also contributed significantly during the driest months of the year [45]. These results confirm the role of Amaranthaceae in extending seasonal aeroallergen exposure across semi-arid landscapes.

The family Urticaceae comprises approximately 1200 species, including widespread genera such as *Urtica* and *Parietaria*. These taxa are typically associated with moist, nitrogen-rich, and disturbed environments and exhibit high ecological adaptability to both urban and natural habitats [74,75]. Among these, *Parietaria* pollen is particularly relevant as a major aeroallergen in Mediterranean and urban regions, where it has been strongly linked to respiratory allergic diseases. Sensitization rates can exceed 70% in southern Europe and continue to rise in densely populated areas [5,74].

In Siirt, Urticaceae accounted for 2.13% of the total airborne pollen and was present in the atmosphere from late March through to the summer months (Table 1 and Figure 5). This extended seasonal activity aligns with findings from Elazığ and Hatay, where Urticaceae contributed consistently to the spring pollen load [18,44]. Its long-duration presence in Siirt suggests a sustained role in background allergenic exposure throughout the vegetative season.

The genus *Quercus* (oak), belonging to the family Fagaceae, comprises over 400 species predominantly distributed across temperate regions of the Northern Hemisphere [76,77]. *Quercus* pollen is considered moderately allergenic and has been implicated in seasonal allergic rhinitis, with occasional cross-reactivity to plant-derived foods [5,72]. In Siirt, *Quercus* represented 1.55% of APIn, with a pronounced peak in April–May. Comparable spring peaks have been reported in Elazığ, where *Quercus* is a minor yet consistent contributor to the atmospheric pollen profile [44].

The family Fabaceae is taxonomically diverse and ecologically significant. While its pollen is generally regarded as having low allergenic potential, it may contribute to background exposure in agricultural and semi-natural environments [5,78]. In Siirt, Fabaceae pollen constituted 1.29% of the total, exhibiting a prolonged season from April to September (Table 1 and Figure 5). Similar patterns have been observed in Hatay and Mardin, where Fabaceae appear sporadically throughout spring and summer [16,18].

*Rumex*, a member of the Polygonaceae family, is a common component of grasslands and disturbed habitats, and produces allergenic pollen primarily in spring [78,79]. In Siirt, *Rumex* accounted for 1.02% of the total airborne pollen, with a pollen season extending from late March to early June. This short-duration peak aligns with data from Elazığ, reflecting its typical early-flowering phenology in semi-arid climates [44].

The timing and duration of the MPS in Siirt reflect the combined influence of regional floristic composition and prevailing climatic conditions. Typically, the pollen season commenced with Cupressaceae/Taxaceae as early as March, followed by Pinaceae, *Platanus*, and *Quercus* in early to mid-spring. These early-flowering arboreal taxa contributed to a sharp increase in total pollen concentrations during April, which consistently emerged as the peak month in both 2022 and 2023. This temporal pattern mirrors observations from other southeastern Anatolian cities, such as Mardin, Van, and Hatay, where Cupressaceae and other wind-pollinated trees dominate the spring atmospheric pollen profile, often peaking between March and April, depending on local thermal conditions [16,18,43].

As the season progressed, herbaceous taxa, such as Poaceae, Urticaceae, and Amaranthaceae, became increasingly prominent. Poaceae dominated the middle phase of the season, with pollen release extending from late April through to early September. This prolonged MPS is consistent with trends observed in Sarıkamış, Kars, and even Mexico City, where grasses exhibit extended flowering due to staggered germination in both cultivated and ruderal habitats [15,31,34]. Amaranthaceae pollen was most abundant during late summer and early autumn, particularly under warm and arid conditions. Urticaceae displayed the longest atmospheric presence among herbaceous taxa, spanning nearly the entire vegetative period.

Overall, the pollen season in Siirt extended from early March to mid-October, exhibiting a biphasic structure; arboreal taxa predominated from March to May, while herbaceous taxa were dominant from June onward. This seasonal progression is characteristic of semi-arid continental climates and closely aligns with the phenological dynamics reported in Posof, where a similar early–late season dichotomy has been documented [31]. In contrast, coastal Mediterranean cities, such as Antalya and Nicosia, experience earlier onset and prolonged duration of the pollen season, primarily due to milder winters and extended flowering intervals [32,80].

Meteorological factors play a pivotal role in regulating airborne pollen dynamics by influencing plant phenology, pollen maturation, dispersal efficiency, and atmospheric residence time. Among these, temperature is considered one of the most critical drivers, as it accelerates floral development and enhances pollen release, particularly in anemophilous taxa [81,82]. In contrast, elevated relative humidity and precipitation often suppress pollen concentrations by inhibiting anther dehiscence and promoting rain-induced washout or sedimentation of airborne particles [83,84]. Wind speed contributes to the horizontal and vertical dispersion of pollen, facilitating long-distance transport, particularly in open landscapes and for taxa producing small, buoyant grains [85].

In Siirt, statistical correlations between daily meteorological variables and pollen concentrations revealed taxon-specific and seasonally dependent patterns. Temperature exhibited a significant positive correlation with total airborne pollen concentrations across both study years, especially during the flowering periods of Poaceae, Urticaceae, and Amaranthaceae. This supports the established role of thermal conditions in promoting pollen release and enhancing atmospheric persistence. In contrast, relative humidity and precipitation were negatively correlated with pollen levels—most notably during peak periods of Cupressaceae and Poaceae—reflecting the inhibitory effects of moisture on pollen dispersal and the anther-opening process (Table 3).

These meteorological–pollen interactions are consistent with findings from other regions of Türkiye. For instance, in Mardin, pollen concentrations of Cupressaceae and Amaranthaceae were significantly reduced during rainy spring episodes. In Gümüşhane, increased spring precipitation coincided with lower airborne levels of *Alnus* and *Betula* [16]. Internationally, similar moisture-driven suppression of pollen concentrations has been observed in Salamanca (Spain) and Funchal (Portugal), where Cupressaceae and *Olea* pollen loads declined during humid conditions [35,64].

Interestingly, wind speed exhibited weak and inconsistent correlations with daily pollen concentrations in Siirt. This may be attributed to the region’s relatively enclosed topography and low variability in wind direction and intensity. Nonetheless, short-term wind events may still facilitate local pollen dispersal, particularly for herbaceous taxa such as Poaceae and Urticaceae. In contrast, in cities, like Trabzon, Konya, and Trieste, wind has been identified as a more dominant factor influencing pollen transport and vertical stratification. Collectively, these findings underscore the dominant influence of temperature and precipitation on aeropalynological dynamics in Siirt and highlight the importance of integrating meteorological variability in airborne pollen forecasting for semi-arid continental settings (Table 3). Moreover, the observed interannual differences in pollen concentrations may also be partly attributed to variability in these meteorological factors. In our study, the notably higher pollen concentrations recorded in the spring of 2022 compared to 2023 may be explained by the higher mean temperature in April 2022 (17.4 °C) compared to April 2023 (14.5 °C), which likely facilitated more intense flowering and pollen release, particularly among dominant woody taxa. In a 38-year study conducted in Sweden, a positive correlation was found between increasing spring temperatures and rising *Betula* pollen levels [86]. Other studies have also demonstrated a general upward trend in pollen concentrations over long time periods, suggesting that climate change exerts a significant influence on atmospheric pollen levels and season duration [87,88].

## 5. Conclusions

This study provides the first comprehensive aerobiological assessment of airborne pollen diversity in Siirt, a southeastern Anatolian province characterized by complex topography and a transitional phytogeographical position between Irano-Turanian and Mediterranean zones. During the two-year observation period, a total of 18,666 pollen grains/m^3^ representing 37 airborne taxa were identified, with woody taxa constituting the majority of the total pollen load. The most dominant families—Pinaceae, Cupressaceae/Taxaceae, and Poaceae—reflect both the region’s native vegetation and anthropogenic land-use patterns.

Clear seasonal variation was observed, with peak concentrations recorded in April and minimal values during the winter months. The incorporation of CORINE land cover data further revealed that natural grasslands and degraded forest areas represent key source habitats for the recorded pollen spectrum. These findings emphasize the value of integrating ecological landscape data in interpreting aerobiological patterns.

Spearman correlation analysis revealed statistically significant associations between daily pollen concentrations and key meteorological drivers, particularly temperature, relative humidity, and precipitation. These results confirm the sensitivity of pollen emissions and atmospheric persistence to short-term climatic fluctuations, highlighting the need for continuous monitoring in the context of increasing climate variability.

Taken together, the findings of this study provide a valuable baseline dataset for southeastern Türkiye, facilitating the development of regional pollen calendars and supporting public health initiatives for allergy-sensitive populations. Future studies should extend the monitoring duration and incorporate phenological observations and long-term climate datasets to better elucidate ecological responses to environmental change.

## Figures and Tables

**Table 1:** Pollen taxa and associated taxa identified in the atmosphere of Siirt in 2022 and 2023 with mean values and percentages.

Taxa	2022		2023		Mean	
	Total (p/m^3^)	%	Total (p/m^3^)	%	Total (p/m^3^)	%
**Pinaceae**	**3394**	**33.97**	**2392**	**27.57**	**2893**	**31.00**
**Cupressaceae/Taxaceae**	**2932**	**29.35**	**2255**	**25.99**	**2594**	**27.79**
**Moraceae**	**434**	**4.34**	**356**	**4.10**	**395**	**4.23**
***Quercus***	**169**	**1.69**	**120**	**1.38**	**145**	**1.55**
*Platanus*	84	0.84	89	1.03	87	0.93
*Acer*	70	0.70	85	0.98	78	0.83
*Fraxinus*	80	0.80	72	0.83	76	0.81
Rosaceae	66	0.66	67	0.77	67	0.71
*Pistacia*	50	0.50	62	0.71	56	0.60
*Salix*	27	0.27	74	0.85	51	0.54
*Olea*	44	0.44	49	0.56	47	0.50
*Juglans*	41	0.41	51	0.59	46	0.49
*Ligustrum*	21	0.21	26	0.30	24	0.25
*Populus*	23	0.23	18	0.21	21	0.22
*Betula*	8	0.08	8	0.09	8	0.09
*Alnus*	7	0.07	7	0.08	7	0.08
Ericaceae	1	0.01	3	0.03	2	0.02
*Tilia*	1	0.01	3	0.03	2	0.02
*Fagus*	1	0.01	-	-	1	0.01
*Ulmus*	1	0.01	-	-	1	0.01
**Woody taxa**	**7454**	**74.61**	**5737**	**66.12**	**6596**	**70.67**
**Poaceae**	**1519**	**15.21**	**1920**	**22.13**	**1720**	**18.42**
**Amaranthaceae**	**207**	**2.07**	**245**	**2.82**	**226**	**2.42**
**Urticaceae**	**206**	**2.06**	**191**	**2.20**	**199**	**2.13**
**Fabaceae**	**130**	**1.30**	**110**	**1.27**	**120**	**1.29**
***Rumex***	**93**	**0.93**	**97**	**1.12**	**95**	**1.02**
*Taraxacum*	65	0.65	90	1.04	78	0.83
Boraginaceae	51	0.51	49	0.56	50	0.54
Asteraceae	44	0.44	45	0.52	45	0.48
*Artemisia*	29	0.29	56	0.65	43	0.46
*Mercurialis*	54	0.54	24	0.28	39	0.42
Apiaceae	36	0.36	38	0.44	37	0.40
*Plantago*	25	0.25	27	0.31	26	0.28
Brassicaceae	30	0.30	13	0.15	22	0.23
Lamiaceae	22	0.22	16	0.18	19	0.20
Cyperaceae	16	0.16	9	0.10	13	0.13
*Xanthium*	6	0.06	6	0.07	6	0.06
Caryophyllaceae	3	0.03	3	0.03	3	0.03
**Herbaceous taxa**	**2536**	**25.39**	**2939**	**33.88**	**2738**	**29.33**
**Total**	**9990**	**100.00**	**8676**	**100.00**	**9333**	**100.00**

**Table 2:** Characteristics of the MPS for dominant airborne taxa in Siirt: onset and end dates; duration (days); and peak daily concentrations (pollen/m^3^) in 2022 and 2023.

Taxa		2022	2023
Amaranthaceae	MPS	27 May–26 October	24 May–27 October
	Season length (days)	152	156
	Maximum pollen/day	5 pollen/m^3^—23 September	5 pollen/m^3^—18 September
Cupressaceae/Taxaceae	MPS	20 February–26 May	24 February–31 May
	Season length (days)	95	96
	Maximum pollen/day	177 pollen/m^3^—20 February	134 pollen/m^3^—13 March
Fabaceae	MPS	19 May–2 October	30 April–18 September
	Season length (days)	136	141
	Maximum pollen/day	5 pollen/m^3^—25 June	7 pollen/m^3^—18 June
Moraceae	MPS	5 April–21 May	8 April–29 April
	Season length (days)	46	21
	Maximum pollen/day	74 pollen/m^3^—17 April	56 pollen/m^3^—21 April
Pinaceae	MPS	1 April–30 Jun	5 April–7 Aug
	Season length (days)	90	124
	Maximum pollen/day	484 pollen/m^3^—3 April	395 pollen/m^3^—7 April
Poaceae	MPS	12 April–16 October	18 April–9 October
	Season length (days)	187	174
	Maximum pollen/day	69 pollen/m^3^—17 May	87 pollen/m^3^—17 May
*Quercus*	MPS	31 March–24 May	4 April–29 May
	Season length (days)	54	55
	Maximum pollen/day	12 pollen/m^3^—16 May	10 pollen/m^3^—22 April
*Rumex*	MPS	25 March–20 July	29 March–8 June
	Season length (days)	117	71
	Maximum pollen/day	5 pollen/m^3^—28 May	7 pollen/m^3^—23 May
Urticaceae	MPS	26 March–30 July	30 March–3 June
	Season length (days)	126	65
	Maximum pollen/day	8 pollen/m^3^—27 May	10 pollen/m^3^—20 May

**Table 3:** Spearman correlation coefficients between daily airborne pollen concentrations of dominant taxa and meteorological parameters in Siirt during the full years (2022–2023) and their respective MPS.

	Mean Daily Temperature °C			Mean Daily Relative Humidity (%)			Mean Daily Wind Speed (m/s)			Daily Rainfall (mm)		
	2022	2023	MPS Periods	2022	2023	MPS Periods	2022	2023	MPS Periods	2022	2023	MPS Periods
Amarantaceae	−0.161	−0.008	−0.160	0.171	−0.028	0.189 *	−0.001	−0.177 *	−0.152	0.363	0.334	0.253
Cupressaceae/Tax.	−0.206 *	−0.274 **	−0.211 *	0.197 *	0.339 **	−0.005	−0.142	−0.054	−0.096	−0.033	−0.349 *	−0.022
Fabaceae	−0.176	0.016	−0.130	0.185	−0.041	0.143	0.100	0.113	0.144	−0.211	0.083	−0.293
Moraceae	0.186	0.305	0.131	−0.370 **	−0.176	−0.363 *	−0.294 *	0.031	−0.282	−0.480	−0.165	−0.160
Pinaceae	−0.101	−0.157 *	−0.799 **	0.123	0.163 *	0.766 **	0.024	0.296 **	−0.095	−0.181	−0.363 *	−0.048
Poaceae	−0.014	−0.096	−0.336 **	0.077	0.133	0.405 **	0.299 **	0.336 **	0.322 **	−0.141	−0.222	−0.116
*Quercus*	0.163	−0.059	0.232	0.085	−0.094	−0.128	0.012	−0.028	0.029	−0.106	−0.466	−0.021
*Rumex*	0.011	0.330 *	0.114	0.010	−0.225	−0.052	−0.146	0.247	−0.016	−0.423	−0.197	−0.086
Urticaceae	−0.198	0.179	−0.399 **	0.092	−0.058	0.381 **	−0.085	−0.086	−0.185	−0.161	−0.076	0.064

**. Correlation is significant at the 0.01 level (2-tailed). *. Correlation is significant at the 0.05 level (2-tailed). Notes: Statistically significant correlations are shown in bold.

## Data Availability

The original contributions presented in this study are included in the article. Further inquiries can be directed at the corresponding author.

## Abbreviations

The following abbreviations are used in this manuscript:

APIn	Annual Pollen Integral
CORINE	Coordination of Information on the Environment
MPS	Main Pollen Season

## References

[1] Scott R.J., Spielman M., Dickinson H.G. (2004). Stamen structure and function. Plant Cell.

[2] Williams J., Mazer S. (2016). Pollen-tiny and ephemeral but not forgotten: New ideas on their ecology and evolution. Am. J. Bot..

[3] Moore P.D., Webb J.A., Collinson M.E. (1991). Pollen Analysis.

[4] Buters J.T.M., Antunes C., Galveias A., Bergmann K.C., Thibaudon M., Galán C., Schmidt-Weber C., Oteros J. (2018). Pollen and spore monitoring in the world. Clin. Transl. Allergy.

[5] D’Amato G., Cecchi L., Bonini S., Nunes C., Annesi-Maesano I., Behrendt H., Liccardi G., Popov T., Van Cauwenberge P. (2007). Allergenic pollen and pollen allergy in Europe. Allergy.

[6] D’Amato G., Chong-Neto H.J., Ortega O.P.M., Vitale C., Ansotegui I., Rosario N., Haahtela T., Galan C., Pawankar R., Murrieta-Aguttes M. (2020). The effects of climate change on respiratory allergy and asthma induced by pollen and mold allergens. Allergy Eur. J. Allergy Clin. Immunol..

[7] Traidl-Hoffmann C. (2022). Pollen on their way astray—First contact via cross-kingdom signaling leading to far-reaching consequences for the atopic march. Allergy.

[8] Rodríguez-Rajo F., Méndez J., Jato V. (2005). Factors affecting pollination ecology of *Quercus* anemophilous species in North-West Spain. Bot. J. Linn. Soc..

[9] Negrini A., Negrini S., Giunta V., Quaglini S., Ciprandi G. (2011). Thirty-year survey on airborne pollen concentrations in Genoa, Italy: Relationship with sensitizations, meteorological data, and air pollution. Am. J. Rhinol. Allergy.

[10] Gregory P.H. (1978). Distribution of airborne pollen and spores and their long distance transport. Pure Appl. Geophys..

[11] Hedhly A., Hormaza J., Herrero M. (2005). Influence of genotype-temperature interaction on pollen performance. J. Evol. Biol..

[12] Jetschni J., Fritsch M., Jochner-Oette S. (2023). How does pollen production of allergenic species differ between urban and rural environments?. Int. J. Biometeorol..

[13] Çeter T., Özler H., Pınar N.M. (2020). First aeropalynological survey on the atmosphere of Sinop, Turkey. Kastamonu Univ. J. For. Fac..

[14] Kızılpınar İ., Doğan C., Artaç H., Reisli İ., Pekcan S. (2012). Pollen grains in the atmosphere of Konya (Turkey) and their relationship with meteorological factors, in 2008. Turk. J. Bot..

[15] Karadağ G.E.A., Altunoğlu M.K. (2024). Airborne pollen seasonality of Kars province, a high-altitude region in NE Anatolia-Turkey. Palynology.

[16] Tosunoglu A., Saatcioglu G., Bekil S., Malyer H., Bicakci A. (2018). Atmospheric pollen spectrum in Stone City, Mardin; the Northern Border of Mesopotamia/SE-Turkey. Environ. Monit. Assess..

[17] Güvensen A., Öztürk M. (2002). Airborne pollen calendar of Buca-İzmir, Turkey. Aerobiologia.

[18] Tosunoğlu A., İlçim A., Malyer H., Bıçakçı A. (2018). Aeropalynological spectrum of Hatay, Turkey, the eastern coast of the Mediterranean Sea. Aerobiologia.

[19] Celenk S., Bicakci A., Tamay Z., Guler N., Altunoglu M.K., Canitez Y., Malyer H., Sapan N., Ones U. (2010). Airborne pollen in European and Asian parts of Istanbul. Environ. Monit. Assess..

[20] Bıçakçı A., Tosunoğlu A. (2019). Allergenic pollen in Turkey. Asthma Allergy Immunol..

[21] Pınar S.M., Fidan M., Eroğlu H. (2021). Siirt ili florasına genel bir bakış. Commagene J. Biol..

[22] Özyazıcı M.A., Dengiz O., İmamoğlu A. (2014). Siirt ili bazı arazi ve toprak özelliklerinin coğrafi bilgi sistem analizleriyle değerlendirilmesi. Türkiye Tarımsal Araştırmalar Derg..

[23] Mut S. (2020). Comparative Land Use in the Central District of Siirt and Tillo District. Master’s Thesis.

[24] Galán C., Cariñanos P., Alcázar P., Dominguez-Vilches E. (2007). Spanish aerobiology network (REA) management andquality manual. Serv. Publicaciones Univ. Córdoba.

[25] Andersen T.B. (1991). A model to predict the beginning of the pollen season. Grana.

[26] de Weger L.A., Bergmann K.C., Rantio-Lehtimäki A., Dahl Å., Buters J., Déchamp C., Belmonte J., Thibaudon M., Cecchi L., Besancenot J.P., Sofiev M., Bergmann K.-C. (2013). Impact of pollen. Allergenic Pollen: A Review of the Production, Release, Distribution and Health Impacts.

[27] American Academy of Allergy, Asthma & Immunology (n.d.) AAAAI: The Global Leader in Allergy, Asthma, and Immunology. http://www.aaaai.org/.

[28] Tasioulis T., Karatzas K., Charalampopoulos A., Damialis A., Vokou D. (2022). Five ways to define a pollen season: Exploring congruence and disparity in its attributes and their long-term trends. Aerobiologia.

[29] Spieksma F.T.M., D’Amato G., Spieksma F.T.M., Bonini S. (1991). Regional European pollen calendars. Allergenic Pollen and Pollinosis in Europe.

[30] Karabağ M. (2023). Determination of Atmospheric Pollens in Ardahan Province, Posof District. Master’s Thesis.

[31] Akpınar S., Altunoğlu M.K. (2024). Determination of atmospheric pollen grains by volumetric method in Sarıkamış District (Kars-Türkiye). Biology.

[32] Gucel S., Guvensen A., Ozturk M., Çelik A. (2013). Analysis of airborne pollen fall in Nicosia (Cyprus). Environ. Monit. Assess..

[33] Rizzi-Longo L., Pizzulin-Sauli M., Stravisi F., Ganis P. (2007). Airborne pollen calendar for Trieste (Italy), 1990–2004. Grana.

[34] Calderón-Ezquerro M.C., Guerrero-Guerra C., Martínez-López B., Fuentes-Rojas F., Téllez-Unzueta F., López-Espinoza E.D., Calderón-Segura M.E., Martínez-Arroyo A., Trigo-Pérez M.M. (2016). First airborne pollen calendar for Mexico City and its relationship with bioclimatic factors. Aerobiologia.

[35] Camacho I.C. (2015). Airborne pollen in Funchal City, (Madeira Island, Portugal)-First pollinic calendar and allergic risk assessment. Ann. Agric. Environ. Med..

[36] Jiang K., Du C., Huang L., Luo J., Liu T., Huang S. (2023). Phylotranscriptomics and evolution of key genes for terpene biosynthesis in Pinaceae. Front. Plant Sci..

[37] He T., Belcher C., Lamont B., Lim S. (2015). A 350-million-year legacy of fire adaptation among conifers. J. Ecol..

[38] Bowe L.M., Coat G., de Pamphilis C.W. (2000). Phylogeny of seed plants based on all three genomic compartments: Extant gymnosperms are monophyletic and Gnetales’ closest relatives are conifers. Proc. Natl. Acad. Sci. USA.

[39] Krutovsky K.V., Troggio M., Brown G.R., Jermstad K.D., Neale D.B. (2004). Comparative mapping in the Pinaceae. Genetics.

[40] Domínguez-Ortega J., López-Matas M., Alonso M., Felíu A., Ruiz-Hornillos J., González E., Moya R., Carnés J. (2016). Prevalence of allergic sensitization to conifer pollen in a high *Cypress* exposure area. Allergy Rhinol..

[41] Erkara İ., Cingi C., Ayrancı Ü., Gürbüz K., Pehlivan S., Tokur S. (2008). Skin prick test reactivity in allergic rhinitis patients to airborne pollens. Environ. Monit. Assess..

[42] García-Gallardo M.V., Algorta J., Longo N., Espinel S., Aragones A., Lombardero M., Bernaola G., Jauregui I., Aranzabal A., Albizu M.V. (2013). Evaluation of the effect of pollution and fungal disease on *Pinus radiata* pollen allergenicity. Int. Arch. Allergy Immunol..

[43] Bicakci A., Tosunoglu A., Altunoglu M.K., Saatcioglu G., Keser A.M., Ozgokce F. (2017). An aeropalynological survey in the city of Van, a high altitudinal region, East Anatolia-Turkey. Aerobiologia.

[44] Kilic M., Altunoglu M.K., Akpınar S., Akdogan G.E., Taskin E. (2019). Relationship between airborne pollen and skin prick test results in Elazığ, Turkey. Aerobiologia.

[45] Kafashan H.A., Khosravi A.R., Alyasin S., Sepahi N., Kanannejad Z., Shirazi F.M.A.Z., Karami S. (2021). Airborne pollens and their association with meteorological parameters in the atmosphere of Shiraz, Southwest Iran. Iran. J. Allergy Asthma Immunol..

[46] Yang Y., Yang Z., Ferguson D.K., Shong J.Y. (2023). An integrative view on the systematic position of the cupressophyte Cephalotaxus. Ecol. Evol..

[47] Ghimire B., Jeong M., Lee C., Heo K. (2018). Inclusion of *Cephalotaxus* in Taxaceae: Evidence from morphology and anatomy. Korean J. Pl. Taxon..

[48] Quinn C., Price R., Gadek P. (2002). Familial concepts and relationships in the conifer based on rbcL and matK sequence comparisons. Kew Bull..

[49] Majeed A., Singh A., Choudhary S., Bhardwaj P. (2019). RNAseq-based phylogenetic reconstruction of Taxaceae and Cephalotaxaceae. Cladistics.

[50] Sundararaj R., Mathimaran A., Prabhu D., Ramachandran B., Jeyaraman J., Muthupandian S., Asmelash T. (2024). In silico approaches for the identification of potential allergens among hypothetical proteins from *Alternaria alternata* and its functional annotation. Sci. Rep..

[51] Galveias A., Costa A.R., Bortoli D., Alpizar-Jara R., Salgado R., Costa M.J., Antunes C.M. (2021). Cupressaceae pollen in the city of Évora, South of Portugal: Disruption of the pollen during air transport facilitates allergen exposure. Forests.

[52] Charpin D., Pichot C., Belmonte J., Sutra J.P., Zidkova J., Chavez P., Shahali Y., Senéchal H., Poncet P. (2017). *Cypress* pollinosis: From tree to clinic. Chin. Rev. Allergy Immunol..

[53] Yoo K.H., Kwon T.R., Kim Y.U., Kim E.H., Kim B.J. (2020). The effects of fabric containing *Chamaecyparis obtusa* essential oil on atopic dermatitis-like lesions: A functional clothing possibility. Ski. Pharmacol. Physiol..

[54] Majrashi A.A. (2022). Preliminary assessment of weed population in vegetable and fruit farms of Taif, Saudi Arabia. Braz. J. Biol..

[55] Arthan W., Baker W.J., Barrett M.D., Barrett R.L., Bennetzen J., Besnard G., Bianconi M.E., Birch J.L., Catalán P., Grass Phylogeny Working Group III (2024). Nuclear phylogenomics of grasses (Poaceae) supports current classification and reveals repeated reticulation. bioRxiv.

[56] Adachukwu O., Kenneth E., Chukwu O., Adaugo N., Chisom I. (2023). Ecological survey on species of Poaceae family present in Nnamdi Azikiwe University campus Awka, Anambra State. Asian J. Environ. Ecol..

[57] Céccoli G., Ramos J., Pilatti V., Dellaferrera I., Tivano J.C., Taleisnik E., Vegetti A.C. (2015). Salt glands in the Poaceae family and their relationship to salinity tolerance. Bot. Rev..

[58] Xu X., Dimitrov D., Shrestha N., Rahbek C., Wang Z. (2021). Sensitization profiles of timothy grass pollen in Northern China. J. Asthma Allergy.

[59] Li J.D., Gu J.Q., Xu Y.Y., Cui L., Li L.S., Wang Z.X., Yin J., Guan K. (2022). Serum IgE profiles in Chinese pollinosis patients with grass pollen sensitization. World Allergy Organ. J..

[60] Mohamed M., Refaat M., Melek N., Ahmed E., Aldin N., Latif O. (2022). Pollen sensitization among Egyptian patients with respiratory allergic diseases. Egypt. J. Immunol..

[61] Puc M. (2010). Threat of allergenic airborne grass pollen in Szczecin, Nw Poland: The dynamics of pollen seasons, effect of meteorological variables and air pollution. Aerobiologia.

[62] Kmenta M., Bastl K., Berger U., Kramer M.F., Heath M.D., Pätsi S., Pessi A.-M., Saarto A., Werchan B., Werchan M. (2017). The grass pollen season 2015: A proof of concept multi-approach study in three different European cities. World Allergy Organ. J..

[63] Celenk S., Bicakci A. (2005). Aerobiological investigation in Bitlis, Turkey. Ann. Agric. Environ. Med..

[64] Rodríguez-de la Cruz D., Sánchez-Reyes E., Dávila-González I., Lorente-Toledano F., Sánchez-Sánchez J. (2010). Airborne pollen calendar of Salamanca, Spain, 2000–2007. Allergol. Immunopathol..

[65] Clement W.L., Weiblen G.D. (2009). Morphological evolution in the Mulberry family (Moraceae). Syst. Bot..

[66] Aneklaphakij C., Bunsupa S., Sirichamorn Y., Bongcheewin B., Satitpatipan V. (2020). Taxonomic notes on the ‘Mahat’ (*Artocarpus lacucha* and *A. thailandicus*, Moraceae) species complex in Thailand. Plants.

[67] Papia F., Incorvaia C., Genovese L., Gangemi S., Minciullo P.L. (2020). Allergic reactions to genus *Morus* plants: A review. Clin. Mol. Allergy.

[68] Ahmed F. (2021). Ficus benghalensis bark extract shows blood pressure lowering effect in normotensive and angiotensin II-induced hypertensive rats. Pharmacophore.

[69] Li J.D., Du Z.R., Liu J., Xu Y.Y., Wang R.Q., Yin J. (2020). Characteristics of pollen-related food allergy based on individual pollen allergy profiles in the Chinese population. World Allergy Organ. J..

[70] Nitiu D.S. (2006). Aeropalynologic analysis of La Plata City (Argentina) during a 3-year period. Aerobiologia.

[71] Tosunoğlu A., Bıçakçı A., Malyer H., Sapan N. (2009). Analysis of airborne pollen fall in Köyceğiz specialty protected area (SW Turkey). Fresenius Environ. Bull..

[72] Gharbi D., Al-Nesf M., Trigo M. (2022). Do we need aerobiological air monitoring in desert climates? The Qatar experience. Qatar Med. J..

[73] Piotrowska-Weryszko K., Weryszko-Chmielewska E., Sulborska A., Puc M., Malkiewicz M., Siergiejko Z., Dąbrowska-Zapart K., Ziemianin M., Rapiejko A., Wieczorkiewicz A. (2020). Concentration of pollen of Chenopodiaceae/Amaranthaceae plants in the air of selected Polish cities in 2020. Alergoprofil.

[74] Gonçalves F., Sousa A., Oliveira R. (2023). Use of mass spectrometry as a tool for the search or identification of flavonoids in Urticaceae. Rodriguésia.

[75] Arilla M.C., González-Rioja R., Ibarrola I., Mir A., Monteseirín J., Conde J., Martínez A., Asturias J.A. (2006). A sensitive monoclonal antibody-based enzyme-linked ımmunosorbent assay to quantify *Parietaria judaica* major allergens, Par j 1 and Par j 2. Clin. Exp. Allergy.

[76] Cavender-Bares J. (2018). Diversification, adaptation, and community assembly of the American oaks (*Quercus*), a model clade for integrating ecology and evolution. New Phytol..

[77] Hipp A.L., Manos P.S., Hahn M., Avishai M., Bodénès C., Cavender-Bares J., Crowl A.A., Deng M., Denk T., Fitz-Gibbon S. (2020). Genomic landscape of the global oak phylogeny. New Phytol..

[78] Li J.-J., Li Y.-X., Li N., Zhu H.-T., Wang D., Zhang Y.-J. (2022). The genus *Rumex* (Polygonaceae): An ethnobotanical, phytochemical and pharmacological review. Nat. Prod. Bioprospect..

[79] Tommaso M., Luciani A., Crisi P., Beschi M., Rosi P., Rocconi F., Miglio A. (2021). Detection of serum allergen-specific ige in atopic dogs tested in Northern Italy: Preliminary study. Animals.

[80] Tosunoglu A., Altunoglu M.K., Bicakci A., Kilic O., Gonca T., Yilmazer I., Saatcioglu G., Akkaya A., Celenk S., Canitez Y. (2015). Atmospheric pollen concentrations in Antalya, South Turkey. Aerobiologia.

[81] Anderegg W.R.L., Abatzoglou J.T., Anderegg L.D.L., Bielory L., Kinney P.L., Ziska L. (2021). Anthropogenic climate change is worsening North American pollen seasons. Proc. Natl. Acad. Sci. USA.

[82] Ziska L., Knowlton K., Rogers C., Dalan D., Tierney N., Elder M.A., Filley W., Shropshire J., Ford L.B., Hedberg C. (2011). Recent warming by latitude associated with increased length of ragweed pollen season in central North America. Proc. Natl. Acad. Sci. USA.

[83] Harbele S.G., Bowman D.M.J.S., Newham R.M., Johnston F.H., Beggs P.J., Buters J., Campbell B., Erbas B., Godwin I., Green B.J. (2014). The macroecology of airborne pollen in Australia and New Zealand urban areas. PLoS ONE.

[84] Veriankaitė L., Šaulienė I., Bukantis A. (2011). Evaluation of meteorological parameters influence upon pollen spread in the atmosphere. J. Environ. Eng. Landsc..

[85] Izquierdo R., Belmonte J., Avila A., Alarcón M., Cuevas E., Alonso-Pérez S. (2011). Source areas and long-range transport of pollen from continental land to Tenerife (Canary Islands). Int. J. Biometeorol..

[86] Frei T., Gassner E. (2008). Climate change and its impact on birch pollen quantities and the start of the pollen season: An example from Switzerland for the period 1969–2006. Int. J. Biometeorol..

[87] Bortenschlager S., Bortenschlager I. (2005). Altering airborne pollen concentrations due to global warming: A comparative analysis of airborne pollen records from Innsbruck and Obergurgl (Austria) for the period 1980–2001. Grana.

[88] Yli-Panula E., Fekedulegn D.B., Green B.J., Ranta H. (2009). Analysis of airborne Betula pollen in Finland: A 31-year perspective. Int. J. Environ. Res. Public Health.

